# Chemokine receptor CXCR7 non-cell-autonomously controls pontine neuronal migration and nucleus formation

**DOI:** 10.1038/s41598-020-68852-z

**Published:** 2020-07-16

**Authors:** Yan Zhu, Tatsumi Hirata, Fabienne Mackay, Fujio Murakami

**Affiliations:** 1 Graduate School of Frontier Biosciences, Osaka University, Yamadaoka 1-3, Suita, Osaka 565-0871 Japan; 2 School of Biomedical Sciences, The University of Melbourne, Parkville, VIC 3010 Australia; 3 QIMR Berghofer Medical Research Institute, Herston, QLD 4006 Australia; 4 Brain Function Laboratory, National Institute of Genetics, Graduate University for Advanced Studies, SOKENDAI, Yata 1111, Mishima, Shizuoka 411-8540 Japan

**Keywords:** Developmental biology, Neuroscience

## Abstract

Long distance tangential migration transports neurons from their birth places to distant destinations to be incorporated into neuronal circuits. How neuronal migration is guided during these long journeys is still not fully understood. We address this issue by studying the migration of pontine nucleus (PN) neurons in the mouse hindbrain. PN neurons migrate from the lower rhombic lip first anteriorly and then turn ventrally near the trigeminal ganglion root towards the anterior ventral hindbrain. Previously we showed that in mouse depleted of chemokine receptor CXCR4 or its ligand CXCL12, PN neurons make their anterior-to-ventral turn at posteriorized positions. However, the mechanism that spatiotemporally controls the anterior-to-ventral turning is still unclear. Furthermore, the role of CXCR7, the atypical receptor of CXCL12, in pontine migration has yet to be examined. Here, we find that the PN is elongated in Cxcr7 knockout due to a broadened anterior-to-ventral turning positions. Cxcr7 is not expressed in migrating PN neurons *en route* to their destinations, but is strongly expressed in the pial meninges. Neuroepithelium-specific knockout of Cxcr7 does not recapitulate the PN phenotype in Cxcr7 knockout, suggesting that CXCR7 acts non-cell-autonomously possibly from the pial meninges. We show further that CXCR7 regulates pontine migration by modulating CXCL12 protein levels.

## Introduction

During development new born neurons migrate radially or tangentially from the progenitor zones to their final destinations^1–4^. Tangential migration is often deployed by neurons that migrate over long distances, sometimes across brain compartments, to distant destinations^5–7^. Such long-distance tangential migration usually involves multiple changes of migratory directions through varied tissue environments, hence demands a complex set of underlying guidance mechanisms. While much has been learned of the guidance of tangential migration owing in part to its shared mechanisms with the guidance of growing axons^2,8–10^, mechanisms that underlie changes of migratory directions are still incompletely understood.

**Figure 1:**
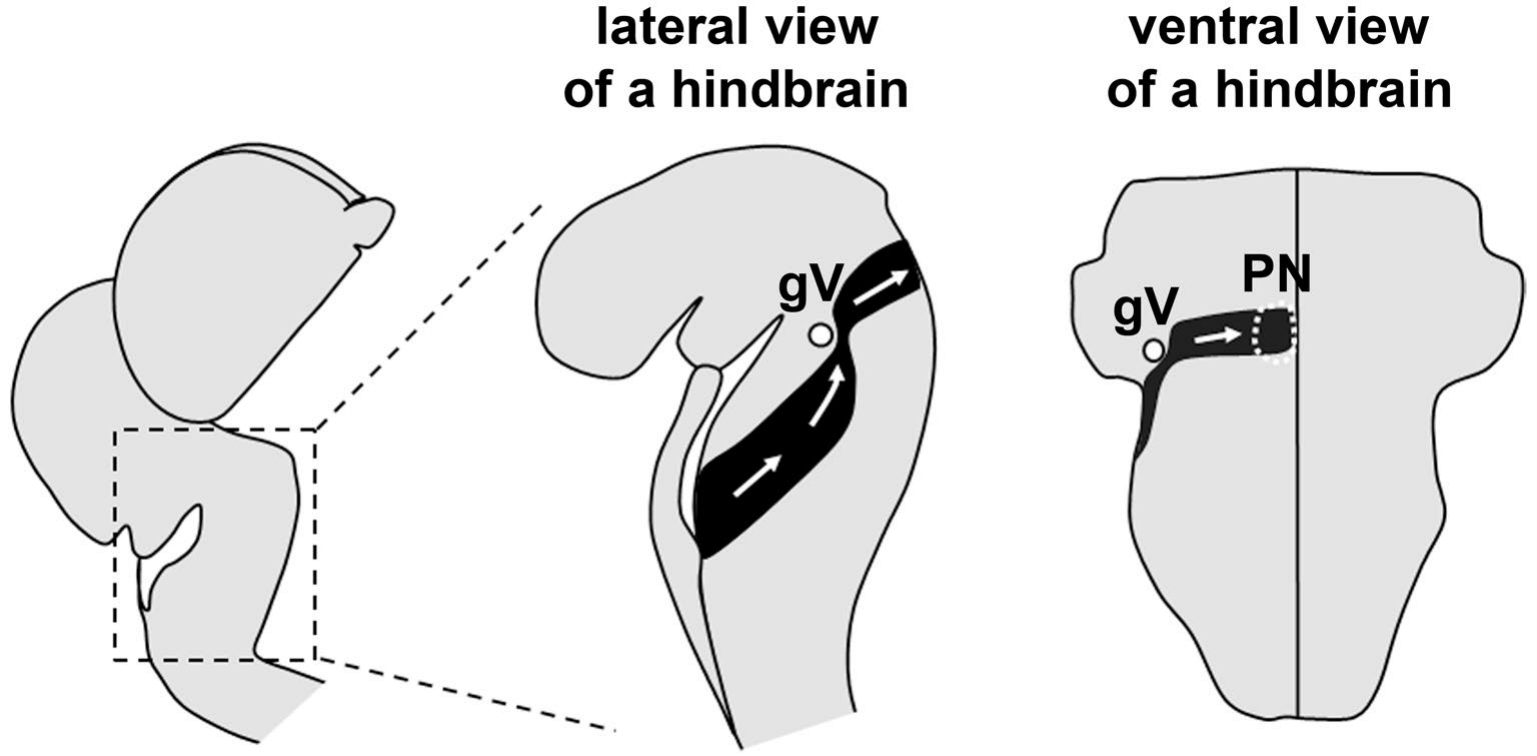
A schematic showing the migratory stream of PN neurons during development. The left panel shows a developing mouse brain at around E15. The middle and the right panel show a lateral and a ventral view of an E15 mouse hindbrain, respectively. The migratory stream of PN neurons on the left side of the brain is indicated in black. White arrows indicate the migratory directions. The small filled circle indicates the position of the root of the trigeminal ganglion (gV). The region circled by white broken line indicates the prospective PN nuclear region.

The migration of PN neurons in the mouse hindbrain is an excellent model to investigate the guidance mechanisms underlying long-distance tangential migration^11–16^. PN neurons are destined to form the pontine nuclei, an amalgamation of pontine gray nucleus and reticular tegmental nucleus, which form a part of the precerebellar system that relays information from the rest of the CNS to the cerebellum^17–19^. These neurons are born from the lower rhombic lip, a progenitor zone lining the dorsal edge of the posterior hindbrain (Fig. 1)^20–24^. They then migrate anteriorly over the pial surface of the hindbrain beneath the pial meninges (Fig. 1)^11,17,25^. At the level of the root of the trigeminal ganglion (gV), anteriorly migrating PN neurons make a sharp turn ventrally, heading orthogonally towards the ventral midline (Fig. 1)^13,26^. Near the ventral midline, most PN neurons detach from the migratory stream without crossing the midline and disseminate into the prospective ipsilateral pontine nuclear region^11,15,25,27^. We previously showed that a chemokine CXCL12 (also called SDF-1) secreted from the pial meninges and its receptor CXCR4 expressed in the migrating PN neurons regulate two key processes of pontine migration^13^. First, disruption of CXCL12/CXCR4 signalling results in some PN neurons migrating deep in the hindbrain parenchyma without migrating anteriorly, owing to a loss of the chemoattraction that usually confines the pontine migratory stream to the pial surface of the hindbrain. Second, in both Cxcr4 and Cxcl12 mutant mice, many PN neurons that migrate superficially turn ventrally at positions posterior to gV root, suggesting that CXCL12/CXCR4 signalling is important for the anteriorly migrating PN neurons to reach the gV root before turning ventrally. The mechanism of how CXCL12 imposes spatial/temporal regulation on the anterior-to-ventral turning in PN neurons is still unknown.

CXCR7 is a later identified atypical receptor of CXCL12^28,29^ which plays diverse cellular functions, depending on its cellular and tissue context, and biological conditions. It acts either as a scavenger receptor to shape CXCL12 availability and distribution, a modulator of CXCL12 downstream signalling via heterodimerization with CXCR4, or in some cases an active signalling receptor^30,31^. Since the role of CXCR7 has not yet been examined in pontine migration, we reasoned that unravelling the role of CXCR7 might lend clues to this question.

Here, we examined the role of CXCR7 in the migration of PN neurons by analysing its expression pattern and phenotypes in Cxcr7 knockout mice. We find that CXCR7 regulates the anterior-to-ventral turning positions of migrating PN neurons and the shape of the resultant PN. We provide evidence that CXCR7 regulates this process non-cell-autonomously, possibly through controlling CXCL12 protein levels.

## Results

### Expression of Cxcr7 in the hindbrain during PN neuron migration

**Figure 2:**
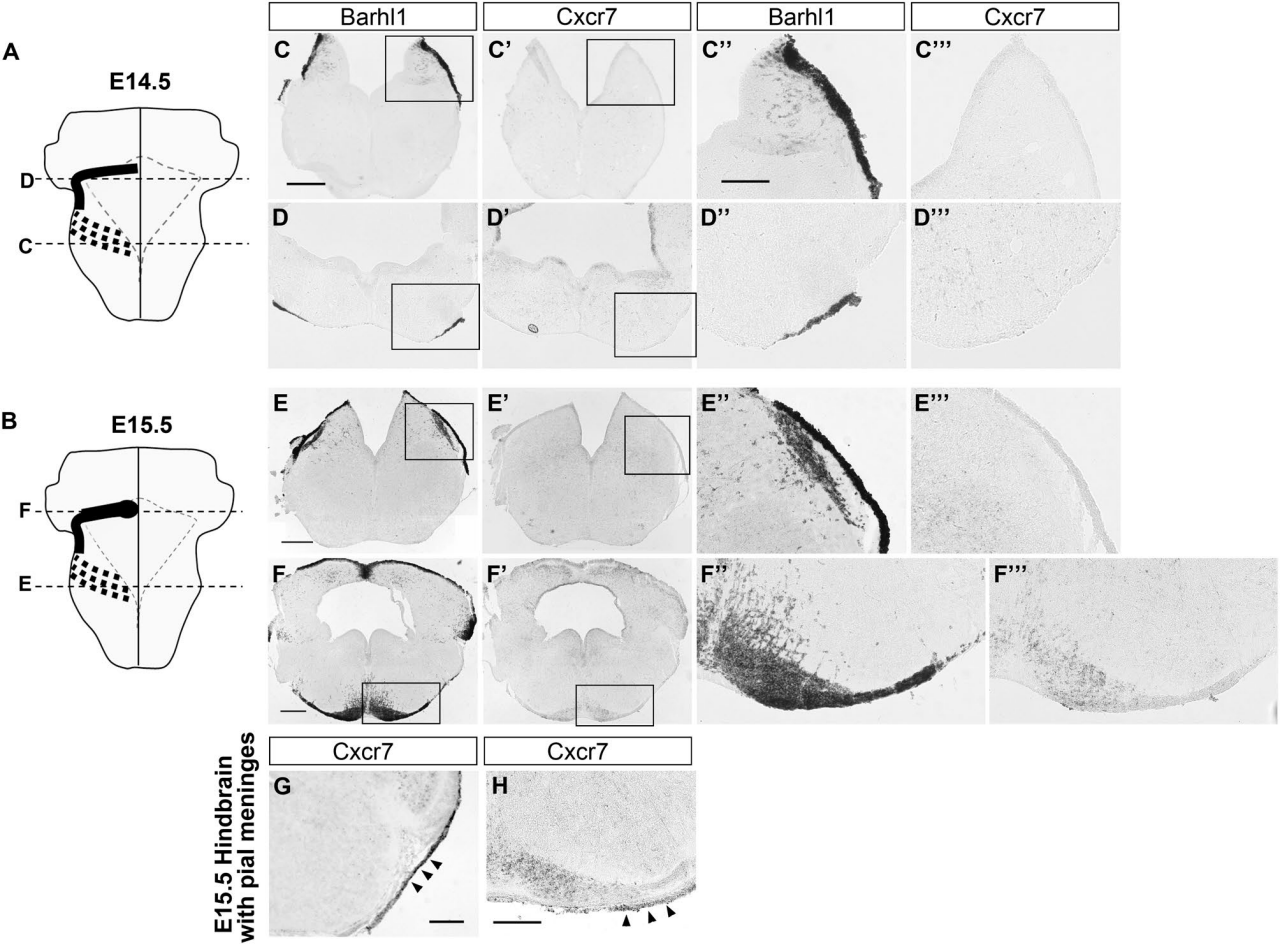
Expression patterns of Cxcr7 during migration of PN neurons. (**A**,**B**) Schematics illustrate the migrating streams of PN neurons on the left of an E14.5 hindbrain (**A**) and an E15.5 hindbrain (**B**) viewing from the ventral side. The rhomboid shape demarcated by dotted lines indicates the outline of the 4th ventricle on the dorsal side of the hindbrain. The thick dotted black lines indicate the migratory stream departing the rhombic lip on the dorsal side. The thick solid line indicates the migratory stream on the ventral side of the hindbrain. Horizontal broken lines indicate the approximate axial levels of the sections shown in **C**–**F** series. (**C**,**D**) Barhl1 ISH signals on sections from an E14.5 hindbrain showing the pontine migratory stream leaving the rhombic lip (**C**) and at the anteroventral turning point (**D**). (**C**′,**D**′) Cxcr7 ISH on adjacent sections to (**C**,**D**), respectively. Cxcr7 is not expressed in migrating PN neurons. (**C**″,**C**‴,**D**″,**D**‴) High magnification images of boxed areas in (**C**,**C**′,**D,D**′), respectively. (**E**,**F**) Barhl1 ISH signals on sections from an E15.5 hindbrain showing the pontine migratory stream leaving the rhombic lip (E) and entering into the prospective pontine nuclear region (**F**). (**E**′,**F**′) Cxcr7 ISH on adjacent sections to (**E**,**F**), respectively. Cxcr7 only becomes moderately expressed in PN neurons after their arrival at the pontine nuclear region. (**E**″,**E**‴,**F**″,**F**‴) High magnification images of boxed area in (**E**,**E**′,**F**,**F**′), respectively. (**G,H**) Cxcr7 ISH on sections of an E15.5 hindbrain which has its overlying pial meninges kept. Cxcr7 signal is detected in the pial meninges (arrowheads). Scale bars: 400 μm for (**C**,**C**′,**D**,**D**′); 200 μm for (**C**″,**C**‴,**D**″,**D**‴,**E**″,**E**‴,**F**″,**F**‴); 400 μm for (**E**,**E**′); 400 μm for (**F**,**F**′); 200 μm for (**G**,**H**).

To investigate whether CXCR7 is involved in the migration of PN neurons, we first examined the expression of Cxcr7 gene by in situ hybridization (ISH) in developing mouse hindbrains at stages when PN neurons migrate towards the future pontine nuclear region (Fig. 2A,B). PN neurons can be identified by their expression of a Bar domain-containing transcription factor Barhl1 (also known as Mbh2) throughout their development^32,33^. We found that Cxcr7 was detected in PN neurons only after their arrival at the PN region but not during their tangential migration (Fig. 2C–C‴, D–D‴, E–E‴, F–F‴). This expression pattern contrasts with that of Cxcr4, the other CXCL12 receptor, which is expressed in PN neurons during migration, but not after reaching the PN region^13^. The above data were obtained by ISH on mouse hindbrains with their pial meninges removed. When we performed ISH of Cxcr7 on hindbrains with pial meninges attached, we found that Cxcr7 was strongly expressed in the pial meninges overlying the developing hindbrains at stages of pontine migration (Fig. 2G,H, arrowheads). Cxcr7 was also expressed in neuronal subsets of the inferior olive nucleus and facial nucleus, subdomains of the ventricular zone and some scattered cells in hindbrain parenchyma (Supplementary Fig. S1 online).

### PN shape is elongated in Cxcr7 knockout mice

**Figure 3:**
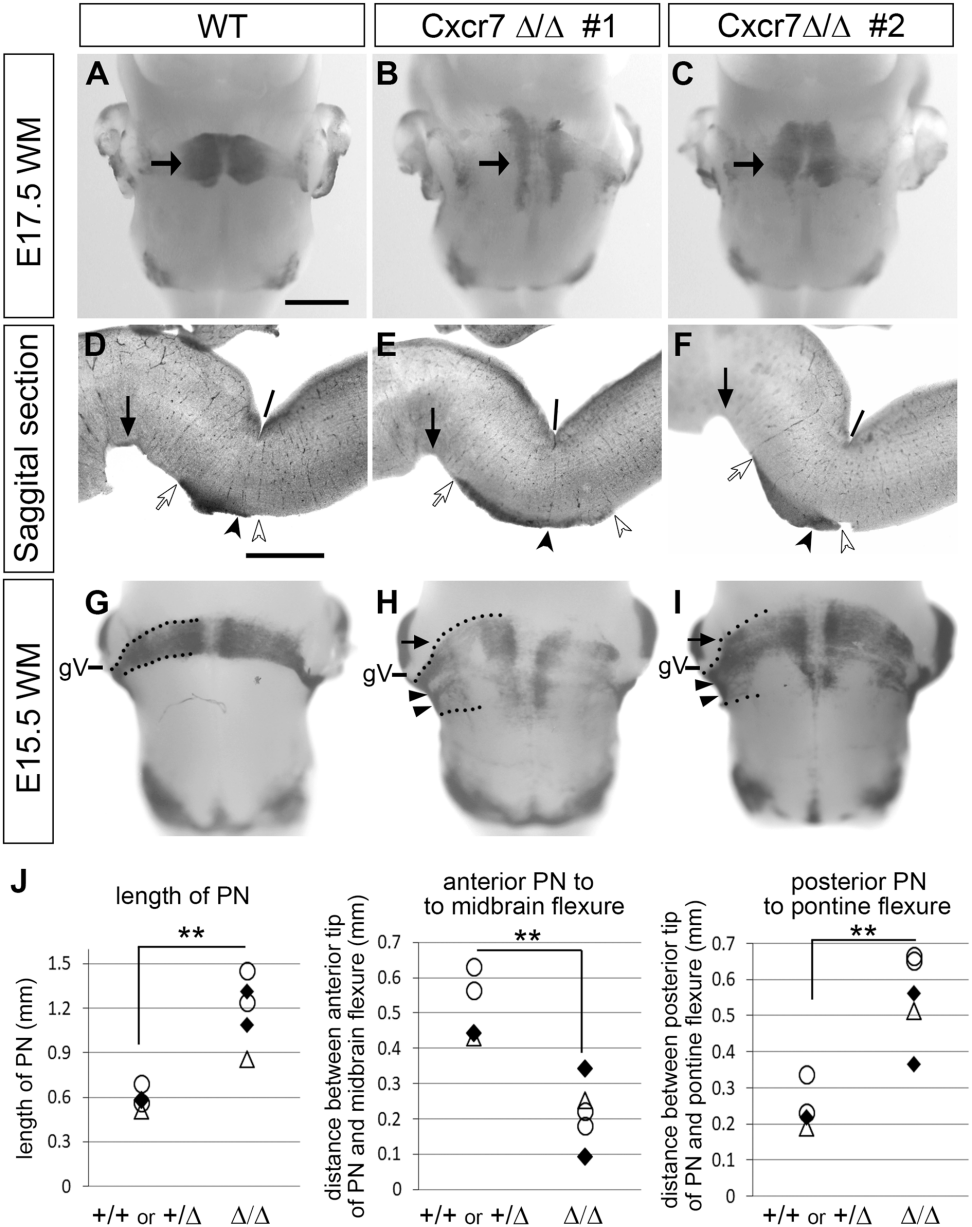
Phenotype analyses of PN formation in Cxcr7 knockout mice. (**A**) Barhl1 ISH on a whole mount (WM) E17.5 wild type (WT) hindbrain. The arrow indicates a prominent PN in the anteroventral hindbrain. (**B**,**C**) Barhl1 ISH on two different WM E17.5 Cxcr7Δ/Δ hindbrains #1 and #2, respectively. Both showed elongated PN along anteroposterior axis, although their severity varied. (**D**–**F**) Parasagittal sections of hindbrains in (**A**–**C**), respectively. Filled arrows indicate the midbrain flexures, lines indicate the pontine flexures, filled arrowheads indicate the points radially opposing the pontine flexures, open arrows indicate the anterior tips of PN, and open arrowheads indicate the posterior tips of PN. Measurements were taken on the sections for quantification (for details, see materials and methods). (**G**,**H,I**) Barhl1 ISH on an E15.5 WM wild type hindbrain (**G**) and two different E15.5WM Cxcr7Δ/Δ hindbrains #1 and #2 (**H,I**). The anteroposterior span of the anterior-to-ventral turning point and the ventrally-directed migratory stream of PN neurons (demarcated by dotted lines) are broader in Cxcr7Δ/Δ than those in the wild type. gV indicates the root of trigeminal ganglion. (**J**) Scatter plots depicting the quantification of PN phenotype between WT/Hetero (n = 4) and Cxcr7Δ/Δ (n = 5). The three scatter plots from left to right show, respectively, the length of the PN, the distance between the anterior tip of the PN and the midbrain flexure, and the distance between the posterior tip of the PN and the pontine flexure. Hindbrains from three independent litters (indicated by circles, triangles and black diamonds, respectively) were used for quantification. Cxcr7Δ/Δ hindbrains have elongated PN (left graph, *p* = 0.0025), which are extended both anteriorly (mid graph, *p* = 0.0028) and posteriorly (right graph, *p* = 0.0026). Statistics were performed with t-tests with unequal variance. Scale bars: 800 μm for (**A**–**C**,**G**–**I**); 600 μm for (**D**–**F**).

We next analyzed PN formation in Cxcr7 knockout mice (Cxcr7Δ/Δ) on whole mount (WM) and sectioned hindbrains. Wild type (WT) or Hetero E17.5 hindbrains showed a prominent PN in the anteroventral hindbrain straddling across the ventral midline shown by ISH (Fig. 3A, n = 4). In Cxcr7Δ/Δ hindbrains, the PN was formed, but appeared to be elongated along the anteroposterior axis (Fig. 3B,C, n = 5). While all mutant hindbrains analyzed showed the elongated phenotype, its severity varied from animal to animal (Fig. 3B, comparing to Fig. 3C) indicating incomplete penetrance. The extent of PN elongation could be better appreciated on parasagittal sections of these hindbrains (Fig. 3D–F). Quantification of the PN elongation showed that the length of PN along anteroposterior axis is longer in Cxcr7Δ/Δ than in WT/Hetero mice (Fig. 3D–F,J). Measurement of the distances between the anterior or posterior tips of PN and morphological hindbrain landmarks indicated that PN in Cxcr7Δ/Δ is elongated both anteriorly and posteriorly (Fig. 3D–F,J).

This phenotype might have been caused by disrupted migration before PN neurons’ arrival at the PN. Alternatively, abnormal anteroposterior movement of PN neurons after arriving at the PN could have caused the phenotype. To distinguish these possibilities, we analyzed pontine migration in Cxcr7Δ/Δ at E15.5, an earlier stage when many PN neurons undergo anterior-to-ventral turning. In wild type, anteriorly migrating PN neurons only started to turn ventrally when they reached the vicinity of gV root (Fig. 3G, n = 3). A confined turning position(s) was represented by all PN neurons executing their ventral turning just before or shortly after they curved around gV root. The resulting ventrally directed migratory stream had its anteroposterior span (demarcated by dotted lines in Fig. 3G) approximately corresponding to that of the PN next to the midline. This is because PN neurons tangentially migrate into the prospective nuclear region with very limited movement along the anteroposterior axis^15^. In Cxcr7Δ/Δ hindbrains, however, the anteriorly migrating PN neurons turned ventrally over a much wider anteroposterior span (Fig. 3H,I, n = 3). Some neurons turned ventrally at posteriorized positions before reaching the gV root (Fig. 3H,I, arrowheads), while some others appeared to migrate further anteriorly after curving around gV root before turning orthogonally towards the ventral midline (Fig. 3H,I, arrows). The dysregulated anterior-to-ventral turning resulted in a sheet of ventrally migrating cells with broader anteroposterior span (demarcated by dotted lines in Fig. 3H,I), which resulted in an elongated PN. Although we cannot completely exclude the possibility that some PN neurons in Cxcr7Δ/Δ hindbrains aberrantly move anteroposteriorly after their arrival at the nuclear region, the fact that the anteroposterior expansion is already present at the anterior-to-ventral turning point in E15.5 Cxcr7Δ/Δ hindbrains suggests that the primary cause for the elongated PN in Cxcr7 knockout mice is due to dysregulated ventral turning of PN neurons.

It should be noted that the PN phenotype in Cxcr7 knockout is distinct from that seen in Cxcr4 (or Cxcl12) knockout mice^13^, in which the mis-positioning of PN neurons occurs entirely posteriorly, but not anteriorly, to the normal PN. In addition, Cxcr4 and Cxcl12 knockout hindbrains show a substantial number of PN neurons migrating deeply and forming a deep ectopic pontine cluster across the midline, which is not observed in Cxcr7 knockout hindbrains.

### Cxcr7 may act non-cell-autonomously in pontine migration

The elongated appearance of the PN was unexpected considering that Cxcr7 was not expressed in PN neurons when they were turning from anterior to ventral direction (Fig. 2D–D‴). We speculated that Cxcr7 might play a non-cell-autonomous role in this phenotype. To address this issue, we generated neuroepithelium-specific conditional Cxcr7 knockout mice using a Nestin-Cre driver line (Nes-Cre) and a Cxcr7 floxed (fl) line. The Nes-Cre line drives Cre-dependent recombination specifically in the developing nervous system from E11 at the latest^34^. Importantly, Nes-Cre line should not affect Cxcr7 expression in the pial meninges of the hindbrain as the meningeal cells around the hindbrains are derived from the mesodermal precursors^35,36^. We first confirmed the specificity of Nes-Cre mediated recombination by crossing Nes-Cre mice with a Cre-responder line Z/EG (Supplementary Fig. S2 online). While Nes-Cre drove recombination throughout the neuroepithelium including the pontine migratory stream, it did not cause recombination in the pial meninges. We then confirmed that Cxcr7 was indeed knocked out from the neuroepithelium including PN, but not from the pial meninges (Supplementary Fig. S3 online). Cxcr7 was expressed in the PN, pial meninges, and some other cells in the hindbrain neuroepithelium as shown by Cxcr7 ISH on E15.5 Cxcr7 fl/Δ sections. Its expression was eliminated in the PN and other neuroepithelium cells, but was preserved in the pial meninges in Nes-Cre:Cxcr7 fl/Δ hindbrains.

**Figure 4:**
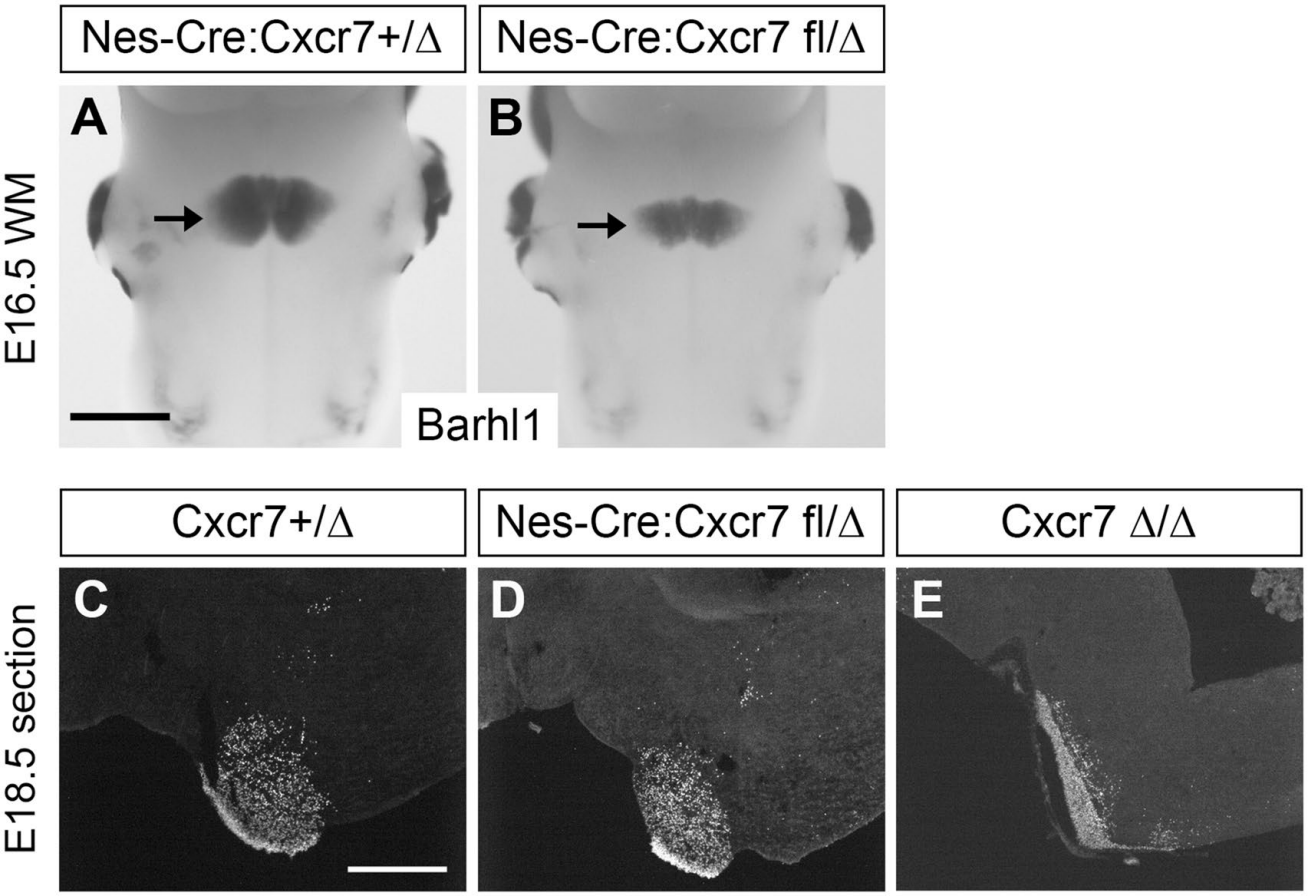
Conditional knockout of Cxcr7 in the neuroepithelium. (**A**,**B**) Barhl1 ISH on E16.5 WM hindbrains of Nes-Cre:Cxcr7 +/Δ, Nes-Cre:Cxcr7 fl/Δ, respectively. The PN is indicated by an arrow. The PN in Nes-Cre:Cxcr7fl/Δ (**B**) does not show the elongated phenotype as in Cxcr7Δ/Δ (see Fig. 3B,C). (**C**,**D**,**E**) BARHL1 immunohistochemistry on parasagittal sections of hindbrains of genotypes Cxcr7 +/Δ, Nes-Cre:Cxcr7 fl/Δ, and Cxcr7Δ/Δ, respectively. The PN in Nes-Cre:Cxcr7 fl/Δ (**D**) does not appear elongated as that in Cxcr7Δ/Δ (**E**). Scale bars: 800 μm for (**A**,**B**); 200 μm for (**C**–**E**).

We then went on to examine the PN phenotype in these conditional knockout mice. We found that the PN was not elongated anteroposterially in Nes-Cre:Cxcr7fl/Δ hindbrains by Barhl1 WM ISH (Fig. 4B) and by BARHL1 immunohistochemistry on parasagittal sections (Fig. 4D) (n = 3). It should be noted that the size of the PN across the anteroposterior and mediolateral dimensions appeared smaller than that in Nes-Cre:Cxcr7 +/Δ (Fig. 4A, n = 2). Comparison of the PN visualized by BARHL1 immunostaining on parasagittal sections showed that the PN in Nes-Cre:Cxcr7fl/Δ (Fig. 4D) did not phenocopy the elongated appearance of PN in Cxcr7Δ/Δ (Fig. 4E), but was similar to, albeit smaller than, the PN in Cxcr7 +/Δ (Fig. 4C). Therefore, knockout of Cxcr7 from neuroepithelial cells including PN neurons did not recapitulate the PN phenotype observed in Cxcr7Δ/Δ, suggesting a non-cell-autonomous role of Cxcr7.

To further substantiate this interpretation, we introduced a Cre expression construct into the rhombic lip of Cxcr7 fl/Δ or Cxcr7 fl/fl embryos by in utero electroporation (IUE) and found that the electroporated PN neurons migrated normally, differing from the electroporated Cxcr7Δ/Δ hindbrains which showed elongated PN (Supplementary Fig. S4 online). These data, taken together, suggest that Cxcr7 acts non-cell-autonomously in regulating PN neuron migration and point to the possibility that CXCR7 expression in the meninges may cause the PN phenotype.

### Meningeal cells endocytose CXCL12 in a CXCR7 dependent manner

**Figure 5:**
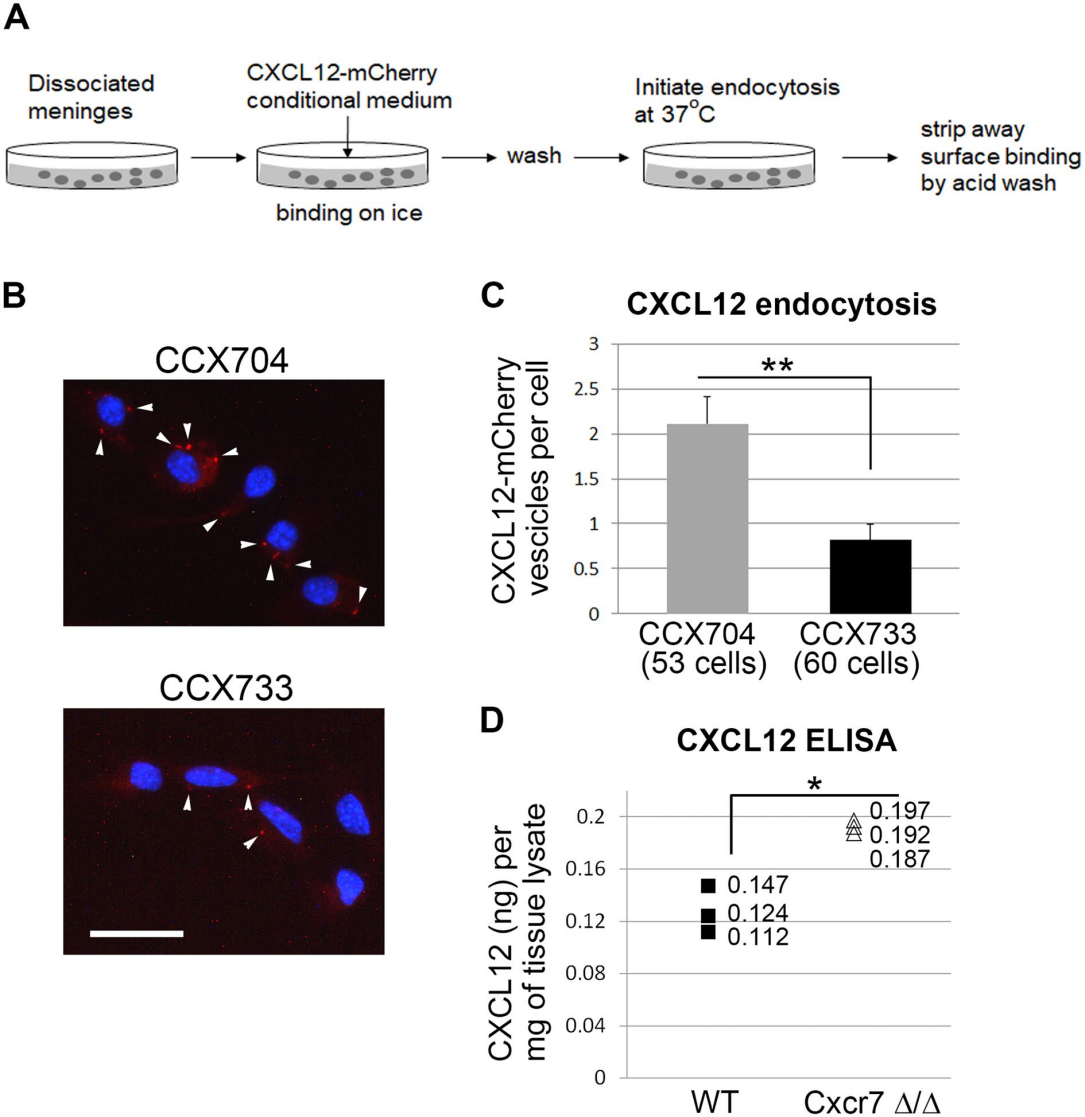
Endocytosis of CXCL12 by meningeal cells and increase of CXCL12 protein levels in Cxcr7 knockout mice. (**A**) A schematic showing the experimental set up of the CXCL12 endocytosis assay on primary culture of dissociated pial meningeal cells. (**B**) Representative images of meningeal cells that were subjected to endocytosis assay. Two conditions were compared: addition of a non-effective control compound (CCX704), and addition of a CXCR7 antagonist (CCX733). CXCL12-mCherry containing vesicle-like particles (indicated by arrowheads) are more abundant in CCX704 than in CCX773. (**C**) CXCL12-mCherry positive vesicles were quantified and the average numbers per cell in CCX704 (from 53 cells) and CCX733 (from 60 cells) were compared in the histogram. The difference between these two conditions is statistically significant (**p = 0.00086, Mann–Whitney U test). The result is represented as mean + SEM. (**D**) CXCL12 ELISA on tissue lysates obtained from WT and Cxcr7Δ/Δ hindbrains with pial meninges attached. Three samples of each genotype were measured for their CXCL12 levels by ELISA. CXCL12 (ng) per mg of tissue lysate for each sample was plotted. CXCL12 levels were on average 1.5 fold higher in Cxcr7Δ/Δ hindbrains than in WT (*p = 0.01844, t-tests with unequal variance). Scale bar: 25 μm for (**B**).

Our results so far raised the possibility that CXCR7 expressed in the meninges plays a non-cell-autonomous role to regulate pontine migration. CXCR7 was previously shown to serve as a scavenger receptor to modulate the ambient CXCL12 levels, which in turn regulates the CXCR4 expressing cells^37–41^. To test whether CXCR7 expressed in the pial meninges might also act as a scavenger of CXCL12, we first examined whether meningeal cells are able to endocytose CXCL12 via their expression of CXCR7. For this, we performed endocytosis assay using a primary culture of pial meningeal cells from embryonic mouse hindbrains and incubated these cells with a culture medium conditioned with CXCL12-mCherry (Fig. 5A). The endocytosis assay was performed in the presence of either CCX733, a selective non-peptide CXCR7 antagonist, or CCX704, a control compound structurally related to CCX733 but with no binding capacity to CXCR7^40,42^. Dissociated meningeal cells from hindbrains were indeed able to endocytose CXCL12-mCherry as evidenced by the presence of mCherry-positive particles in cytoplasmic structures resembling intracellular vesicles such as endosomes (Fig. 5B, CCX704). Addition of CCX733 caused over a 50% reduction of the number of CXCL12-mCherry containing vesicles per cell (Fig. 5B,C). These results suggest that hindbrain meningeal cells are able to endocytose CXCL12 dependent on CXCR7 expression.

### CXCL12 ELISA shows an increased CXCL12 level in Cxcr7Δ/Δ hindbrains

If CXCR7 serves as a scavenger receptor of CXCL12, then an increased level of CXCL12 protein should be expected in CXCR7 knockout hindbrains. To test this, we measured the amount of CXCL12 protein in the wild type and Cxcr7Δ/Δ hindbrain lysates using CXCL12 ELISA. During development, CXCL12 is mainly secreted by the meninges^13,43^. Hindbrains with the pial meninges attached were used to prepare lysates (n = 3 for each genotype). As expected, Cxcr7Δ/Δ showed a higher CXCL12 level than the wild type, with a 1.5 fold increase on average (Fig. 5D). The increase in CXCL12 protein levels is unlikely to be due to a change in Cxcl12 mRNA in CXCR7 deficient mice because Cxcl12 ISH showed comparable mRNA levels and localization between Cxcr7+/Δ and Cxcr7Δ/Δ (Supplementary Fig. S5 online). These results highly suggest that CXCR7 modulates CXCL12 as a scavenger. It should be noted that increases in CXCL12 levels in Cxcr7Δ/Δ hindbrains did not change the cell surface expression of CXCR4 in the migrating PN neurons (Supplementary Fig. S6 online).

### Over-expression of full length Cxcr7 disrupts pontine migration similarly to Cxcr4 or Cxcl12 knockout mice

**Figure 6:**
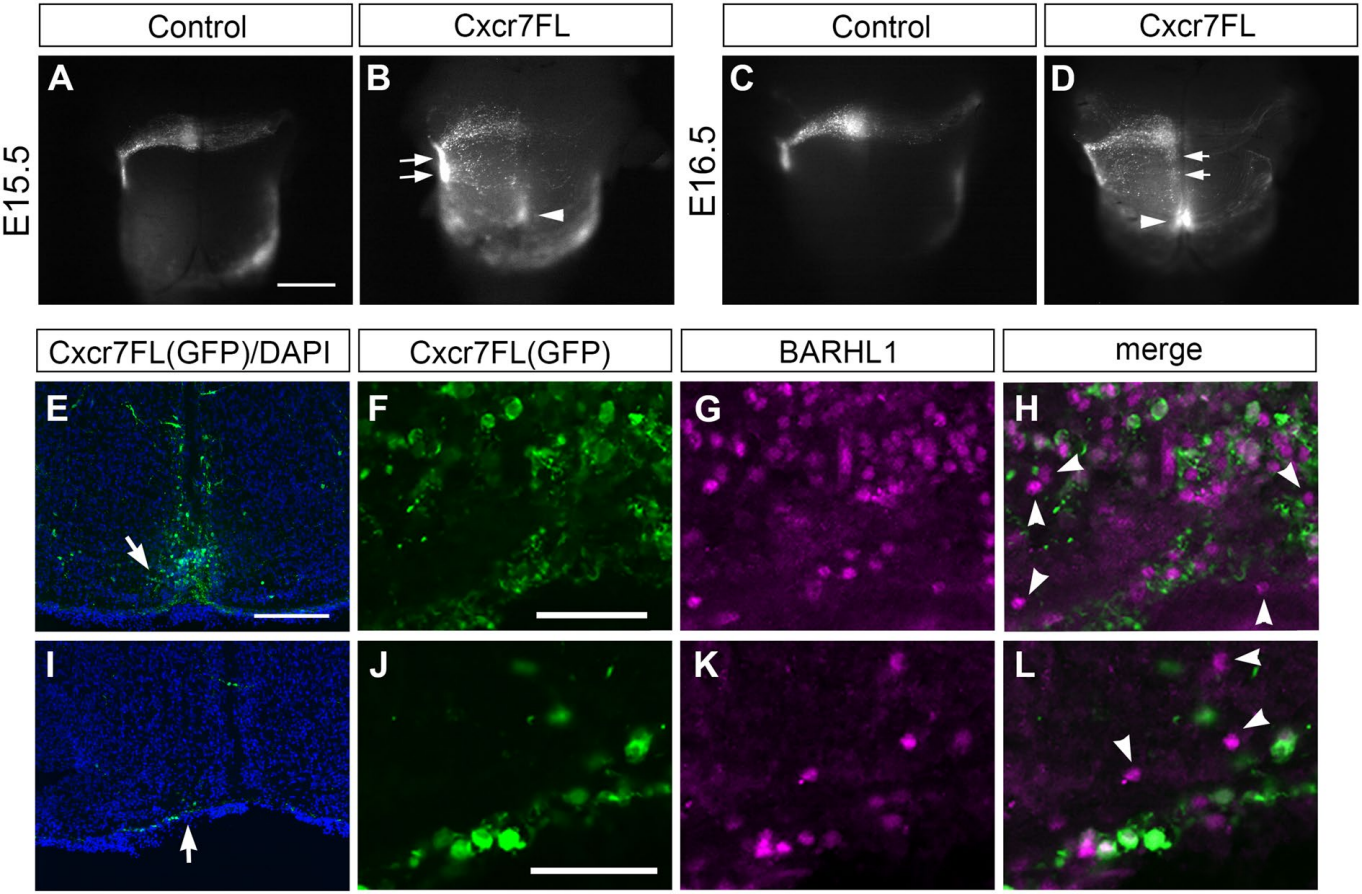
Defects in pontine migration caused by over-expression of full length Cxcr7. (**A**–**D**) Expression constructs were electroporated in utero into the rhombic lip of E12.5 mice hindbrains. pCAGGS-EGFP either alone (**A**,**C**) or together with pCAGGS-Cxcr7FL (**B**,**D**) were introduced and the pontine migratory streams analysed at E15.5 (**A**,**B**) and E16.5 (**C**,**D**). Electroporation of pCAGGS-EGFP alone labelled the pontine migratory streams at E15.5 (**A**) and E16.5 (**C**). Cxcr7FL expression caused disrupted pontine migratory streams (**B**,**D**). Some CXCR7 expressing PN neurons turned at ectopic positions posterior to the normal turning point and formed superficial ectopic clusters (arrows in **B**,**D**), and some formed a deep cluster (out of focus) in a further posterior position across the ventral midline (arrowheads in **B**,**D**). These defects resemble those seen in Cxcr4 or Cxcl12 mutant hindbrains. (**E**) A coronal section of the deep ectopic cluster as indicated by arrowhead in (**D**). CXCR7-expressing neurons formed a cluster away from the pial surface and sat on each side of the ventral midline. (**I**) A coronal section of a superficial ectopic cluster as indicated by arrows in (D). Higher magnification of the areas indicated by arrows in (**E**,**I**) with BARHL1 and GFP double immunostaining are shown in (**F**–**H**,**J**–**L**), respectively. Many BARHL1-positive but GFP-negative PN neurons are located in these ectopic pontine clusters, indicating a non-cell-autonomous effect of CXCR7 over-expression. Scale bars: 800 μm for (**A**–**D**), 200 μm for (**E**,**I**), 25 μm for (**F**–**H**,**J**–**L**).

If endogenous CXCR7 regulates pontine migration by keeping an appropriate level of CXCL12, then an over-expression of full length Cxcr7 in migrating PN neurons might reduce further CXCL12 levels, and result in a pontine migration phenotype resembling that of Cxcl12 or Cxcr4 knockout mice. To test this hypothesis, we co-electroporated a full length Cxcr7 and an EGFP expression constructs into the rhombic lip at E12.5^11,13^. Observation at E15.5 showed that many CXCR7-positive (indicated by GFP-positive) PN neurons took anterior-to-ventral turnings at positions posterior to the normal turning point (Fig. 6B, arrows, compare to 6A, n = 6). Furthermore, a deep (out of focus) cluster across the ventral midline appeared in a posterior position (arrowhead in Fig. 6B). At E16.5, we observed several ectopic superficial CXCR7-positive clusters posterior to the normal PN (Fig. 6D, arrows, compare to 6C, n = 3) and a deep (out of focus) ectopic cluster across ventral midline in a further posterior position (Fig. 6D, arrowhead, n = 3). These two defects phenocopy the two characteristic defects previously observed in Cxcr4 or Cxcl12 knockout mice^13^, suggesting that CXCR7 overexpression compromises CXCR4/CXCL12 signalling.

If over-expressed CXCR7 modulated the ambient CXCL12 levels, then we would expect the introduced CXCR7 exerting a non-cell-autonomous effect. We tested this prediction by immunostaining coronal sections of E16.5 Cxcr7-electroporated hindbrains with GFP and BARHL1 antibodies to visualise electroporated and all PN neurons, respectively. Within the deep ectopic cluster (Fig. 6E,F,G,H) as well as a superficial ectopic cluster (Fig. 6I,J,K,L), we could observe a notable number of BARHL1-positive but GFP-negative cells (arrows) within these mal-positioned PN neuron clusters, suggesting a non-cell-autonomous role of overexpressed CXCR7. These results lend support to the idea that CXCR7 regulates pontine migration via modulating CXCL12 levels.

## Discussion

This study has uncovered that CXCR7 plays an important role in PN formation by regulating the anterior-to-ventral turning of the migrating PN neurons and consequentially the shape of PN. From the expression pattern of CXCR7 and tissue specific conditional CXCR7 knockout, we conclude that CXCR7 acts non-cell-autonomously most likely from the pial meninges. We provided further evidence that CXCR7 may play its role in pontine migration by modulating CXCL12 protein levels via endocytosis. This study has extended our previous work on CXCL12/CXCR4 signalling in pontine migration by revealing a mechanism by which the CXCL12 chemokine concentration is modulated to achieve the precise guidance of the changes in migratory directions during pontine migration.

The PN phenotype in Cxcr7 knockout mice is distinct from that in Cxcr4 (or Cxcl12) knockout mice^13^. There are two notable differences: (1) The PN in Cxcr7 knockout mice elongates beyond both the anterior and posterior limits of the normal PN, whereas in Cxcr4/Cxcl12 knockout mice ectopic pontine clusters are all posteriorized; (2) In Cxcr4/Cxcl12 knockout mice, a portion of PN neurons fail to migrate superficially due to a loss of attraction to the pial meninges. These neurons form a deep posteriorized ectopic cluster. Such a defect is not present in Cxcr7 knockout mice. These differences suggest that depletion of Cxcr7 does not simply compromise CXCL12 signalling.

The elongated PN phenotype in Cxcr7 knockout mice appears to stem largely from a dysregulated earlier event when PN neurons undergo anterior-to-ventral turning around gV. A portion of PN neurons turn prematurely, while another delay turning, contributing to the posteriorized and anteriorized elongation of the PN, respectively. The ectopic turning positions form a continuum with the normal turning position resulting in one elongated PN. This aspect contrasts with the Cxcr4/Cxcl12 knockout mice in which premature turning takes place at a few discrete and sporadic locations leading to separate ectopic clusters. This difference again implies that CXCR7 and CXCR4 act via different mechanisms.

The non-cell-autonomous function of CXCR7 was first implied by Cxcr7 expression patterns, and later evidenced by tissue-specific conditional knockout experiments. Cxcr7 is not expressed at detectable levels in PN neurons throughout their migratory phase. While it is expressed in some other hindbrain neuroepithelial cells, none of them is positioned close enough to influence the migrating PN neurons. By contrast, Cxcr7 is strongly expressed in the pial meninges on which PN neurons migrate. This expression pattern implies that CXCR7 might regulate pontine migration non-cell-autonomously. This possibility is strongly supported by neuroepithelium-specific conditional knockout of Cxcr7. Nestin-Cre mediated recombination reduced Cxcr7 expression to undetectable levels in almost all hindbrain structures that express Cxcr7 without affecting its expression in the pial meninges. The fact that PN in these mice do not show anterior–posterior elongation suggests that CXCR7 expressed from an extra-neural tube source regulates the normal pontine migration. Although direct evidence is still lacking, the pial meninges is highly likely to be this source of CXCR7. The non-cell-autonomous role of Cxcr7 is further supported by the normal migration of PN neurons that were specifically depleted of their Cxcr7 expression via in utero electroporated of Cre. It is intriguing that the PN was smaller in Nes-Cre:Cxcr7fl/Δ hindbrains. The reason for this is still unclear. One possibility is that Cxcr7 expressed in PN neurons after their arrival at the PN region and in other hindbrain structures may regulate the survival and/or production of PN neurons directly or indirectly.

Since the discovery of CXCR7 as the second receptor of CXCL12^28,29^, much effort has been made to understand how CXCR7 interacts with CXCL12/CXCR4 axis in biological systems that are known to utilize CXCL12 signalling^30,31,44^. It has emerged that CXCR7 is largely an atypical receptor of CXCL12 and one of its atypical functions is to act as a scavenger by modulating CXCL12 concentration via CXCR7-dependent CXCL12 endocytosis and subsequent degradation^37,38,40^. Our study suggests that CXCR7 might also serve such a function for the developing pontine system. The expression pattern of Cxcr7 and the neuroepithelium-specific conditional knockout pointed to the importance of CXCR7-expressing pial meninges, which also secrete the ligand CXCL12. We showed that dissociated hindbrain meningeal cells endocytose CXCL12 mainly via CXCR7. CXCR7-mediated CXCL12 endocytosis sends CXCL12 to intracellular degradation pathway^40^, hence clearing CXCL12 from extracellular space and reducing the availability of CXCL12 as a ligand^38,40^. Indeed, we demonstrated a 1.5 fold increase in CXCL12 level in Cxcr7Δ/Δ hindbrain lysate, indicating that CXCR7 serves to keep CXCL12 at a moderate level in hindbrains in vivo. The CXCR7 overexpression experiment lends further support to the scavenger role of CXCR7. An excessive amount of CXCR7 by overexpression generated a pontine migration phenotype resembling mutants with depleted CXCL12 signalling^13^. Two explanations can be envisaged for this phenotype: (1) overexpressed CXCR7 in PN neurons may lead to inhibition of CXCR4 signalling via interaction of or competition between the two receptors^45^, exerting a dominant-negative effect cell-autonomously; (2) an excessive expression of CXCR7 reduces available CXCL12 in the environment via endocytosis-mediated sequestering, which in turn affects pontine migration non-cell-autonomously. Our data showed that an overexpression of CXCR7 affected the migration of both electroporated and non-electroporated PN neurons, hence in favour of the second possibility.

The role of CXCR7 in regulating CXCL12 protein levels available to CXCR4 expressing migrating neurons have been previously demonstrated in mammalian cortical interneurons and Gonadotropin-releasing hormone (GnRH) neurons^39,41,46^. In the former, CXCR7 acts from the cortical interneurons which co-express CXCR7 and CXCR4^39,41^. Whereas in the latter, CXCR7 acts from tissues surrounding the migrating neurons, resembling the scenario presented in this study^46^. Defects in the migration of cortical interneurons and GnRH neurons are thought to be caused mostly by a reduction of cell surface CXCR4 that is triggered by the elevated level of CXCL12 in Cxcr7Δ/Δ^41,46^. However, we do not think this is the case for PN neurons for two reasons. (1) CXCR4 immunoreactivity on the surface of PN neurons was unchanged in Cxcr7 knockout mice (Supplementary Fig. S6 online). (2) The pontine migration phenotype is clearly different between the Cxcr7 and Cxcr4 knockout mice, unlike cortical interneurons and GnHR neurons, whose migration phenotypes were largely similar between these two mutants^39,41,46^. Thus, the pontine phenotype observed in Cxcr7 knockout mice is not due to a reduction of CXCR4 in PN neurons by increased levels of CXCL12. In addition, we found that depletion of CXCR7 did not lead to altered expression of several guidance molecules known to be expressed in migrating PN neurons, such as CNTN-2, DCC and ROBO3 (Supplementary Fig. S7 online). Therefore, we believe that the pontine phenotype in Cxcr7 knockout mice is more likely a consequence of changes in migratory behaviours in direct response to an altered distribution of chemokine as discussed below.

PN neurons turn from anterior to ventral direction within a confined range around the gV root. For the turning to take place, PN neurons would need to lose anteriorly-oriented polarity, and acquire sensitivity to ventrally-directed cues. It has been shown that the ventral migration depends on their expression of receptors DCC and Robo3^47–49^, which collaboratively mediate Netrin signalling^50^. Since anteriorly migrating PN neurons already express DCC and Robo3^48,49^, their responsiveness to Netrin would need to be silenced to prevent premature turning. The phenotype of premature turning has been reported in several mice mutants, including Robo1/2, Slit1/2/3, Phox2A, Unc5C, Ezh2^26,51–53^. At least some of these molecules, including Unc5C and Ezh2, are thought to function via down-regulating the Netrin pathway^51,52^. We have previously reported premature anterior-to-ventral turning of PN neurons in Cxcr4/Cxcl12 mutants^13^. However, how CXCL12 signalling is involved in this process is still unclear. CXCL12 signalling might play a modulatory role in silencing premature responsiveness to Netrin-1 in PN neurons. Alternatively, it may play an instructive role in determining the anterior-oriented polarity of PN neurons via a chemokine gradient. Analysis of Cxcr7 knockout mice in this study showed the intriguing phenotype of both premature and delayed anterior-to-ventral turning. This bimodal defect cannot be easily explained by merely assuming a silencing role of CXCL12, hence pointing to an instructive role of CXCL12 via forming a gradient. We have previously observed a posterior-low anterior-high graded distribution of CXCL12 immunoreactivity along the anterior migratory path of PN neurons^13^. PN neurons might be directed by this gradient to migrate anteriorly until reaching a saturation concentration of CXCL12 at which these neurons could no longer sense the gradient and turn ventrally near gV (Supplementary Fig. S8 online). Such saturation concentration of a chemokine gradient would presumably be caused by full receptor occupancy or receptor desensitization^54–58^. When CXCR7 is depleted, the elevation of CXCL12 concentration might lead to the broadening of the part of the gradient with saturation concentration, which in turn might broaden the anterior-to-ventral turning positions (Supplementary Fig. S8 online). The role of CXCR7 in shaping CXCL12 gradient has been demonstrated in zebrafish, and by using novel fluorescence-based sensors of CXCL12 signalling, CXCL12 gradient and its dynamics have been directly demonstrated in vivo^37,59–61^. Developing similar technologies in mice to enable high resolution and quantitative analyses of the distribution of signalling CXCL12 would be useful to directly address the role of CXCL12 in regulating anterior-to ventral turning of PN neurons.

## Materials and methods

### Animals

The generation of Cxcr7 knockout (Δ) and floxed (fl) mice^62^, Nestin-Cre transgenic line^34^, and Cre responder line Z/EG^63^ have all been described previously. Noon of the day on which a vaginal plug was detected was designated as embryonic day (E) 0.5. For expression studies and in utero electroporation, timed pregnant ICR mice (Nihon SLC, Shizuoka, Japan) were used. In total, 30–35 animals were used for this study. All experimental protocols for animal maintenance, breeding and manipulations were approved by the Institutional Animal Care and Use Committees of Osaka University and National Institute of Genetics, and were conducted in accordance with the Guidelines for the Welfare and Use of Laboratory Animals of the two institutes.

### DNA constructs

The DNA construct for generating Cxcr7 riboprobe for in situ hybridization and the expression constructs pCAGGS-EGFP and pCAGGS-mCherry have been described before^64–67^. A plasmid with nucleotide 103–1,139 of Cxcl12 mRNA (Accession number D21072) was used to generate Cxcl12 riboprobe. pCAGGS-NLS-Cre and pCAGGS-NLS-EGFP are provided by Dr. Yasuto Tanabe (Kyoto University, Japan), and pCALNL5-EGFP by Dr. Kenta Yamauchi (Juntendo University, Japan). The Barhl1 plasmid for generating Barhl1 riboprobe is a kind gift from Dr. Tetsuichiro Saito (Chiba University, Japan). Coding sequence of full length mouse Cxcr7 (Genbank accession: BC015254) was obtained by RT-PCR from total RNA of mouse embryonic brain and subsequently cloned into pCAGGS vector with a multiple cloning site inserted^14,66^ to construct pCAGGS-Cxcr7FL. pCAGGS-Cxcl12-mCherry was cloned by fusing mCherry coding sequences (a kind gift from Dr. Roger Tsien, University of California at San Diego, U.S.A.) to the c-terminus of the mouse Cxcl12 coding sequence (Genbank accession: NM_021704).

### In situ hybridization (ISH) on sections and whole mount (WM) hindbrains

To obtain embryos for ISH, pregnant mice were killed by cervical dislocation and embryos were taken out from the uterus. Mouse hindbrains (E14.5 and E15.5) with or without the overlying pial meninges were dissected out from mouse embryos in phosphate-buffered saline (PBS, pH7.4) and fixed in 4% paraformaldehyde (PFA, 0.1 M PBS) at 4 °C for 6–7 h. The tissues were then cryo-protected in 30% sucrose (in PBS) overnight at 4 °C and embedded in OCT (Sakura FineTek, Japan). Frozen sections were obtained with a cryostat at 20 μm. Hybridizations were performed essentially as previously described^64^. Basically, sections were subjected to proteinase K treatment (1 μg/ml) and post-fixed in 4% PFA for 30 min. Hybridization buffer contained 50% formamide, 1% SDS, 5xSSC (pH 4.5), 50 μg/ml yeast tRNA, 50 μg/ml Heparin in RNase-free water. Riboprobes were used at 1 μg/ml. Hybridization was carried out overnight at 70 °C for Barhl1 riboprobe, and at 65 °C for Cxcr7 riboprobe.

For Barhl1 ISH on WM hindbrains, mouse hindbrains (E15.5, E16.5 and E17.5) with meninges removed were dissected out from mouse embryos and fixed in 4% PFA at 4 °C for overnight. ISH procedure was performed as previously described^13^. Briefly, the fixed hindbrains were permeabilized in 100% methanol. After rehydration, the hindbrains were treated with 10 μg/ml Proteinase K for 20 min and post-fixed in 4% PFA and 0.1% glutaraldehyde. Hybridization buffer contained 50% Formamide, 1.3xSSC (pH 4.5), 5 mM EDTA, 0.5% CHAPS, 50 mg/ml yeast tRNA, 200 μg/ml Heparin and 0.2% Tween-20. Hybridization was carried out with 2 μg/ml Barhl1 riboprobe at 70 °C overnight.

### Immunohistochemistry

Pregnant mice were killed by cervical dislocation and embryos were taken out from the uterus. Hindbrains were dissected out and fixed in 4% PFA at 4 °C for 6–7 h, or overnight for E18.5 hindbrains. Frozen sections were prepared in the same way as those for ISH as described above. Immunohistochemistry was performed as previously described^64^. The sections were blocked with 10% goat or horse serum in PBSTx (0.2% Triton-100) for 1 h followed by incubation with primary antibodies at 4 °C overnight. After washing with PBSTx, the sections were then incubated with the secondary antibodies at room temperature for 2 h. Slides were counter-stained with 0.03% 4,6-diamidino-2-phenylindole (DAPI, Nacalai Tesque). The primary antibodies used were: rabbit anti-BARHL1 (anti-BARHL1) polyclonal antibody (Atlas Antibodies, HPA004809, Sigma, 1:500), chick anti-GFP polyclonal antibody (abcam, ab13970, 1:1,500), rabbit anti-PAX6 polyclonal antibody (Millipore, AB2237, 1:500), rabbit anti-Laminin polyclonal antibody (Sigma, L9393, 1:1,000), goat anti-CXCR4 polyclonal antibody (abcam, ab1670, 1:300), goat anti-DCC polyclonal antibody (Santa Cruz Biotechnology, sc-6535, 1:400), goat anti-ROBO3 polyclonal antibody (R&D systems, AF3076, 1:200) and mouse anti-CNTN2 monoclonal antibody (Developmental Studies Hybridoma Bank, 4D7, 1:50). The secondary antibodies used were cy3-donkey anti-rabbit IgG (Jackson ImmunoResearch, 1:300) for BARHL1 and PAX6 and laminin antibodies, Alexa488-donkey anti-chick IgG (Molecular Probe, Life Technologies, 1:400) for a GFP antibody, cy3-donkey anti-goat IgG (Jackson ImmunoResearch, 1:300) for CXCR4, DCC and ROBO3 antibodies, cy3-goat anti-mouse IgM (Jackson ImmunoResearch, 1:300) for a CNTN2 antibody.

### In utero electroporation

In utero electroporation was performed essentially as previously described^68^ with some modifications. Briefly, pregnant mice were anesthetized with Pentobarbital Sodium (Somnopentyle, Kyoritsu Seiyaku Corporation, Tokyo, Japan, 80 mg/kg body weight). The uterus was exposed after abdominal incision and approximately 2 μl of plasmid (1 mg/ml each of pCAGGS-NLS-EGFP and pCAGGS-Cxcr7FL, or 1 mg/ml each of pCAGGS-NLS-Cre and pCALNL5-EGFP) was injected into the IV ventricle of E12.5 embryos. Five square electric pulses (30 V, 50 ms duration at 100 ms intervals) were applied using a forceps-type electrode (CUY650P5, Nepagene, Japan) connected to a square-pulse generator (CUY21, BEX, Japan).

### Primary culture of dissociated meningeal cells

Pial meninges overlying E13.5 hindbrains from ICR mice were peeled off and torn into small pieces in ice-cold calcium/magnesium free Hanks solution. The meningeal pieces were then treated with 2.5 ml of 0.1% Trypsin at 37 °C for 12 min with occasional rocking. Trypsin reaction was then inhibited by addition of 2.5 ml of DMEM and 10% fetal bovine serum (FBS). Tissues were then washed twice in DMEM-FBS (10%) and dissociated by trituration using a glass Pasteur pipette. Triturated cells were then passed through a 70 μm cell strainer and the cell numbers were counted. Approximately 6 × 10^4^ cells were seeded onto a 12 mm round coverslip placed in a 24 well plate in 500 μl of culture medium comprising DMEM-Glutamine (0.06%)-FBS (10%). Four hours after plating, dead and floating cells were removed and fresh culture medium was added. The dissociated meningeal cells were cultured for 3–4 days before endocytosis assay.

### Preparation of CXCL12-mCherry conditional medium

COS7 cells were transfected with pCAGGS-Cxcl12-mCherry plasmid or pCAGGS-mCherry as a negative control using FuGENE 6 transfection reagent (Promega) and cultured in DMEM-Glutamine-FBS (10%) for 3 days before the conditioned medium were removed for the endocytosis assay.

### Endocytosis assay

Medium of cultured meningeal cells was removed and replaced with DMEM-glutamine without FBS and incubated at 37 °C for 45 min. The cells were then placed on ice and washed twice with cold binding medium (DMEM with 0.1% BSA and 20 mM HEPES). Cxcl12-mCherry conditioned medium (diluted 1:4 in cold binding medium) was laid onto cells, and binding was performed on ice for 2 h. Cells were then washed twice with cold binding medium. The cold binding medium was then replaced with 500 μl of warm culture medium of DMEM-Glutamine-FBS (10%) for each well. One μl of 1 mM CCX773 (ChemoCentryx, Inc., Moutain View, CA, USA) was added to half of the wells, and the other half received 1 μl of 1 mM CCX704 (ChemoCentryx, Inc., Moutain View, CA, USA), so that the final concentration of the added reagent was 2 μM in each well. Endocytosis was then allowed to take place in a 37 °C incubator for 2 h. Cells were then washed twice with cold binding medium. Surface bound Cxcl12-mCherry was stripped by two washes with acid wash buffer (50 mM Sodium Acetate, 150 mM NaCl, pH4.5), 1.5 min each time. The cells were then washed in PBS and fixed in 4% PFA for 20 min at room temperature before DAPI staining and mounting for imaging. Quantification of mCherry positive vesicle-like particles in cells was performed manually.

### Mouse CXCL12 ELISA

E14.5 Cxcr7Δ/ + and Cxcr7Δ/Δ hindbrains with the overlying pial meninges attached were used to prepare tissue lysates. Briefly, hindbrains of desired genotype were quick frozen in liquid nitrogen and stored at -80 °C. On the day of lysate preparation, 200 μl of RIPA buffer (25 mM Tris–HCL pH7.5, 150 mM NaCl, 1% NP-40, 1% Sodium Deoxycholate, 0.1% SDS) with proteinase inhibitor (cOmplete Mini Protease Inhibitor, Roche) was added to each sample, which was then homogenized by a probe type sonicator. Tissue lysate was cleared by centrifugation and the protein quantity was measured by a Pierce BCA Protein Assay Kit (Thermo Scientific) according to the manufacturer’s instruction.

CXCL12 ELISA was performed using a Quantikine ELISA mouse CXCL12 kit (R&D Systems) following the manufacturer’s instruction. Protein concentration of each sample used ranged between 2.5 mg/ml to 3.0 mg/ml. CXCL12 quantity was measured as ng per mg of tissue lysates. Plates were read by an iMark Microplate Absorbance Reader (Bio-Rad).

### Image acquisition and processing

Fluorescence and bright-field images on sections as well as dissociated meningeal cells and fluorescence images on whole mount electroporated hindbrains were captured with a CCD camera (Axiocam, Zeiss) attached to an upright epifluorescence microscope (BX-60, Olympus) at 1,296 × 1,030 pixel resolution. Objective lens used were: 2 × Plan Apo with numerical aperture (NA) 0.08 (Olympus), 4 × UPlan Apo with NA 0.16 (Olympus), 10 × UPlan Apo with NA 0.40 (Olympus) and 20 × UPlan Apo with NA 0.70 (Olympus). Whole hindbrains after ISH were imaged with a Stage Multiviewer System (Keyence) at 1,024 × 1,280 pixel resolution. Adobe Photoshop CS3 or Adobe Photoshop CC were used to adjusted contrast and brightness of images and to assemble figures.

### Quantification of PN elongation

Quantification of PN elongation was performed on parasagittal sections of hindbrains with the PN visualized either by Barhl1 ISH or immunohistochemistry. Three parameters were measured: (1) the length of PN along anterior posterior axis; (2) the distance between the anterior tip of PN and the midbrain flexure and (3) the distance between the posterior tip of PN and the point radially opposite the pontine flexure. Among all sections of a given brain, the sections containing the maximums of parameter (1) and (3) and the minimum of parameter (2) were chosen for comparison between the Cxcr7 WT/Hetero and the knockout mice. Each measurement was normalized for hindbrain size differences using one of the wild type samples as a standard. The distance between the pontine flexure and the midbrain flexure was used as an indicator of the hindbrain size.

### Statistical analysis

Statistical analyses were performed using Prism 8 (GraphPad) and *p* < 0.05 were considered statistically significant.

## Supplementary information

Supplementary file1
