# Supplementary material for: Chemokine receptor CXCR7 non-cell-autonomously controls pontine neuronal migration and nucleus formation
Source: Sci Rep. 2020 Jul 16;10:11830. doi: 10.1038/s41598-020-68852-z (PMC7367352; doi:10.1038/s41598-020-68852-z)

Supplementary information

Title "Chemokine receptor CXCR7 non-cell-autonomously controls pontine neuronal migration and nucleus formation"

Yan Zhu[1, 4, *], Tatsumi Hirata[4], Fabienne Mackay[2, 4], Fujio Murakami[1]

[1]Graduate School of Frontier Biosciences, Osaka University, Yamadaoka 1-3, Suita, Osaka 565-0871, Japan

[2]School of Biomedical Sciences, The University of Melbourne, Victoria 3010, Australia

[3]QIMR Berghofer Medical Research Institute, Queensland 4006, Australia

[4]Brain Function Laboratory, National Institute of Genetics, SOKENDAI, Yata 1111, Mishima, Shizuoka 411-8540, Japan

*corresponding author: yanzhu@nig.ac.jp or yan-zhu@umin.ac.jp

Supplementary figure legend

Supplementary Figure S1 Cxcr7 expression patterns on E15.5 hindbrain sections by ISH. (A), (B) & (C) Three coronal sections of an E15.5 mouse hindbrain from posterior to progressively more anterior positions are shown. Cxcr7 is expressed in the inferior olive nucleus (arrow in (A)) and in subsets of facial nucleus (arrow in (B)). It is also expressed in subdomains of the ventricular zone (arrowhead in (A) and (C)). Cxcr7 is also expressed in scattered neuroepithelial cells as shown in all three sections. Scale bar: 400 μm.

Supplementary Figure S2 Nes-Cre line drives Cre-mediated recombination throughout the neuroepithelium but not in pial meninges. (A), (A)', (A)'' & (A)''' PAX6 and GFP double immunostaining on a coronal section of an E15.5 Nes-Cre/+:Z/EG hindbrain. PAX6 has been previously shown to be a marker of developing PN neurons[1]. GFP expression which resulted from Nes-Cre mediated recombination was found throughout the neuroepithelium including the PAX6-positive migrating PN neurons (arrows in A, A', A'''). (B), (B)', (B)'' & (B)''' Laminin (LAM) and GFP double immunostaining on a coronal section of an E15.5 Nes-Cre/+:Z/EG hindbrain. LAM

expression marks the pial meninges and demarcates the neuroepithlium and the surrounding mesoderm (arrows in B and B'''). Cre-mediated recombination did not take place in the meningeal cells that surround the neural tube. Scale bar: 200 μm.

Supplementary Figure S3 Cxcr7 is selectively knocked out from the neuroepithelium but not from the pial meninges in Nes-Cre:Cxcr7 fl/Δ. (A) & (B) Adjacent coronal sections of an E15.5 Cxcr7fl/Δ hindbrain with Barhl1 and Cxcr7 ISH, respectively. Cxcr7 is expressed in the PN (arrow in B) and the pial meninges (arrowheads in B). (C) & (D) Adjacent sections of an E15.5 Nes-Cre:Cxcr7fl/Δ hindbrain with Barhl1 and Cxcr7 ISH, respectively. Cxcr7 expression is absent from the PN (arrow in D), but maintained in the pial meninges (arrowheads in D). (E) & (F) Dorsal views of coronal sections from an E15.5 Cxcr7fl/Δ hindbrain and an E15.5 Nes-Cre:Cxcr7fl/Δ hindbrain, respectively. Cxcr7 ISH on these sections showed that Cxcr7 expression in the ventricular zone and other neuroepithelial cells were eliminated in Nes-Cre:Cxcr7fl/Δ. Scale bars: 400 μm for (A, B, C, D), 400 μm for (E) and (F).

Supplementary Figure S4 Knockout of Cxcr7 selectively in PN neurons by in utero electroporation (IUE). pCAGGS-NLS-Cre and pCALNL5-EGFP were electroporated into the lower rhombic lip at E12.5. pCAGGS-NLS-Cre expresses a nuclear localization signal (NLS) fused Cre and pCALNL5-EGFP expresses EGFP in a Cre-dependent manner. Labelled PN neurons and PN were visualized at E16.5. (A) IUE into a wild type hindbrain showed pontine migration and the PN. (B) IUE into a Cxcr7 fl/Δ hindbrain showed a pontine migratory stream and the PN similar to the wild type (A). (C) IUE of a pCAGGS-EGFP construct into a Cxcr7Δ/Δ hindbrain showed an elongated PN consistent with the phenotype suggested by Cxcr7 ISH on WM and sections of Cxcr7Δ/Δ hindbrains. Scale bar: 800 μm.

Supplementary Figure S5 Cxcl12 ISH on coronal sections of E14.5 Cxcr7+/Δ and Cxcr7Δ/Δ hindbrains. (A) & (C) are sections from a Cxcr7+/Δ hindbrain; (B) & (D) are sections from a Cxcr7Δ/Δ hindbrain. Sections in (C) & (D) are more rostral than those in

(A) & (B). Cxcl12 mRNA are confined to the pial meninges (arrowheads) and show comparable levels between Cxcr7+/Δ and Cxcr7Δ/Δ. Scale bar: 200 μm

Supplementary Figure S6 CXCR4 expression in migrating PN neurons is similar between WT and Cxcr7Δ/Δ. Coronal sections of an E14.5 WT and an E14.5 Cxcr7Δ/Δ hindbrains were subjected to CXCR4 and PAX6 double immunohistochemistry. PAX6 expression marks the cross section of the anteriorly-migrating pontine stream. (A) & (B): CXCR4; (A') & (B'): PAX6; (A'') & (B''): Merged. CXCR4 expression is comparable between the WT and Cxcr7Δ/Δ. Scale bar: 100 μm.

Supplementary Figure S7 Expression of three guidance molecules known to be expressed in migrating PN neurons are comparable between Cxcr7Δ/+ and Cxcr7Δ/Δ hindbrains. Parasagittal sections of an E16.5 Cxcr7Δ/+ and an E16.5 Cxcr7Δ/Δ hindbrains were subjected to 3 sets of double immunohistochemistry: CNTN2/BARHL1 (A) and (B) series; DCC/BARHL1 (C) and (D) series; and ROBO3/BARHL1 (E) and (F) series. The expression of CNTN2, DCC, and ROBO3 in the pontine migratory stream around the anterio-to-ventral turning point (indicated by an arrow in each image) are comparable between Cxcr7Δ/+ and Cxcr7Δ/Δ. Scale bar: 200 μm.

Supplementary Figure S8 A model based on a CXCL12 gradient in explanation of the PN phenotype observed in Cxcr7Δ/Δ. In this model, the anterior-directed migratory polarity of PN neurons is determined by a posterior-low and anterior-high CXCL12 gradient (blue curve) whose apex correlates with the turning point of pontine migratory stream. We hypothesize a saturation threshold of CXCL12 (vertical dashed black line), above which PN neurons could no longer sense the gradient. Within the region above the saturation threshold (double arrowed blue line), PN neurons lose their anteriorly-directed polarity and turn ventrally (blue rectangle) to form the PN next to the midline (blue oval). In Cxcr7 knockout mice, CXCL12 level is elevated (red curve). Now the region above the saturation threshold extends both anteriorly and posteriorly (double arrowed red line) leading to anteroposterially expanded ventral turning positions (red rectangle) and an elongated PN (red oval).

Supplementary references

1 Engelkamp, D., Rashbass, P., Seawright, A. & van Heyningen, V. Role of Pax6 in development of the cerebellar system. *Development* **126**, 3585-3596 (1999).

Supplementary Fig. 1

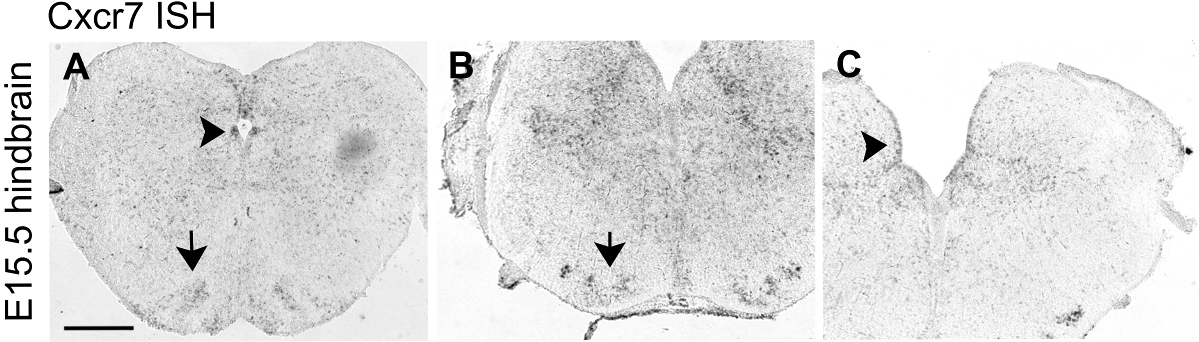

Supplementary Fig. 2

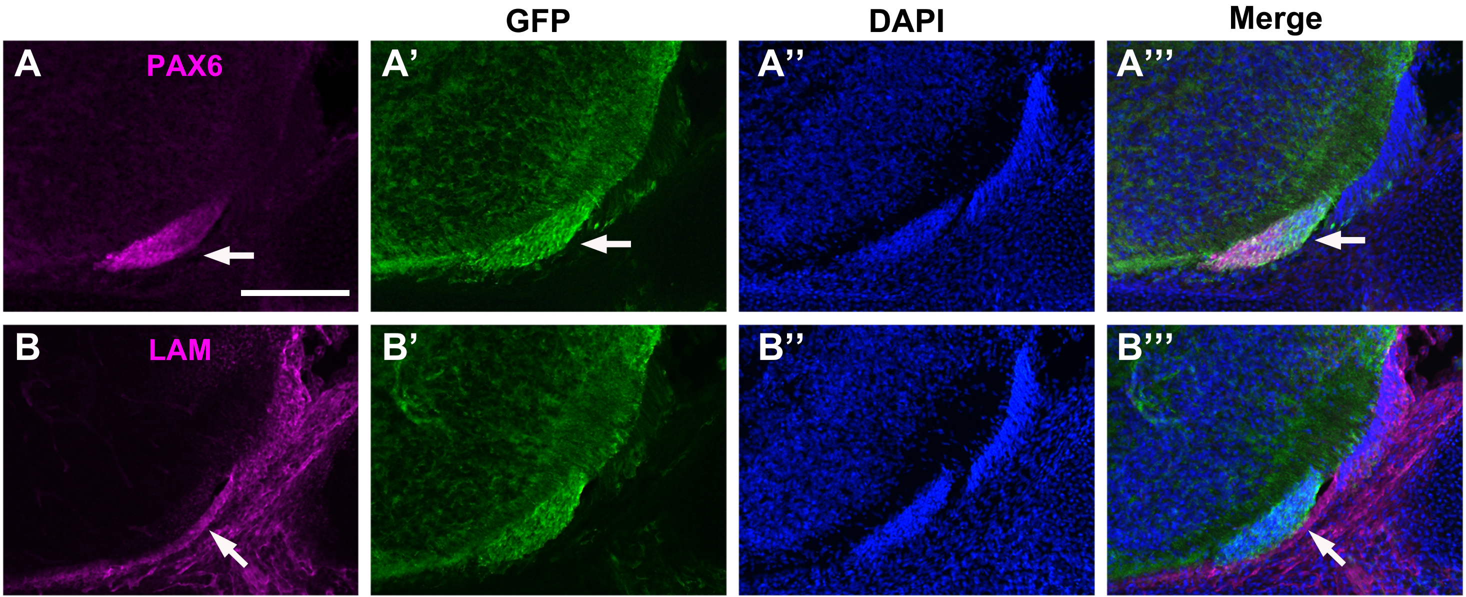

Supplementary Fig. 3

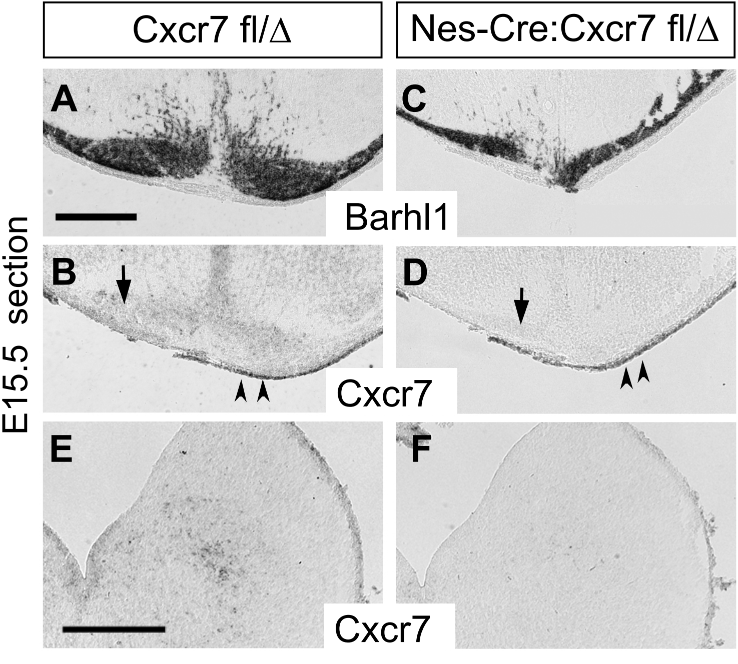

Supplementary Fig. 4

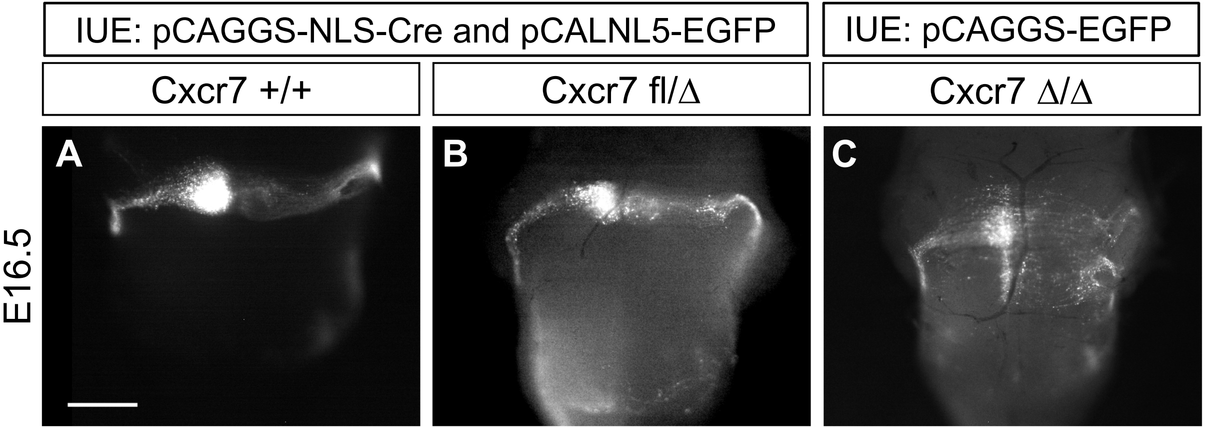

Supplementary Fig. 5

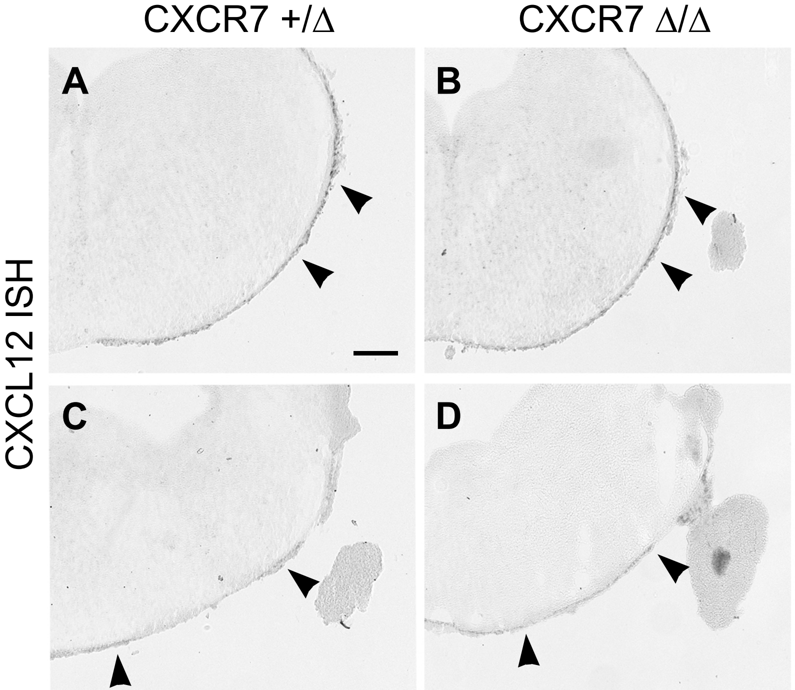

Supplementary Fig. 6

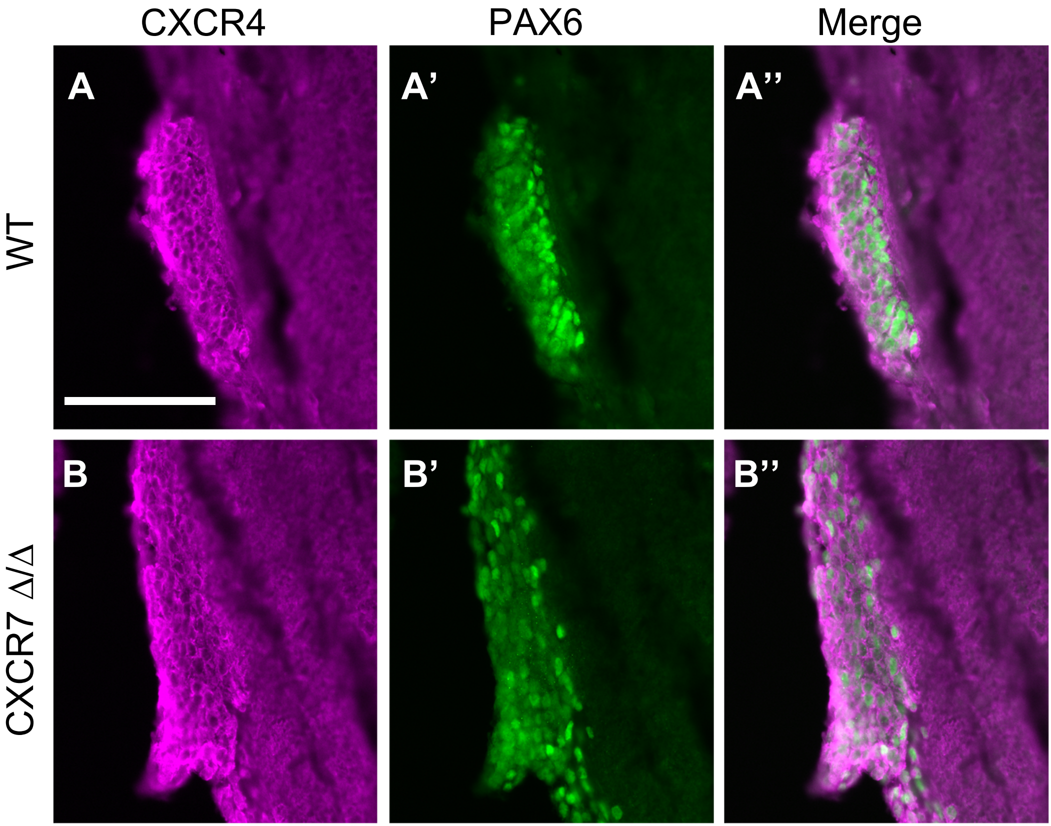

Supplementary Fig. 7

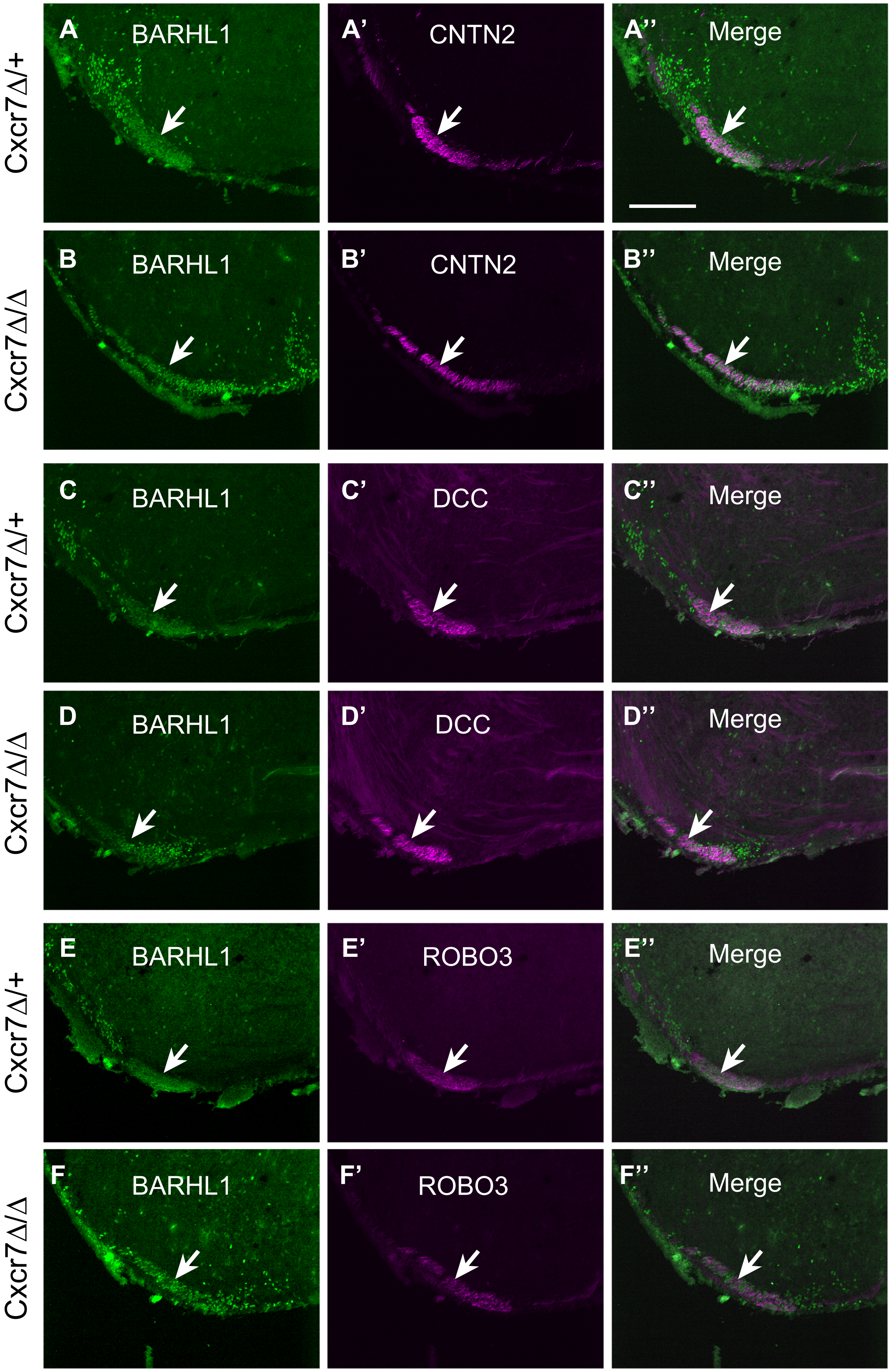

Supplementary Fig. 8

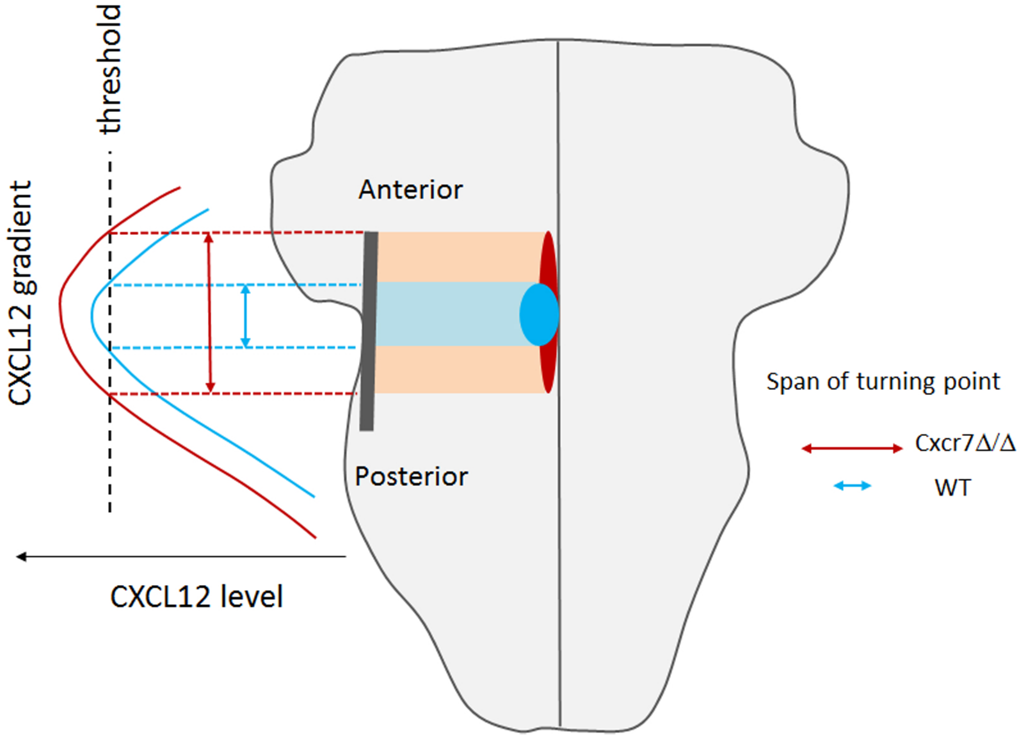

Supplement: Supplementary file 1 — Supplementary file1 [file 41598_2020_68852_MOESM1_ESM.pdf]
